# Challenges to joint planning, monitoring, and evaluation for nutrition-sensitive agriculture in Ethiopia: an exploratory qualitative study

**DOI:** 10.1186/s40066-022-00400-6

**Published:** 2023-01-09

**Authors:** Afework Mulugeta Bezabih, Znabu Hadush Kahsay, Amaha Kahsay, Abate Bekele, Omer Seid, Selemawit Asfaw, Freweeini Gebrearegay, Kidane Tadesse, Alessandra N. Bazzano, Wellington Jogo, Namukolo Covic, Heidi Busse

**Affiliations:** 1 School of Public Health, College of Health Sciences, Mekelle University, Mekelle, Ethiopia; 2 Department of Public Health Nutrition, School of Public Health, College of Medicine and Health Science, Bahir Dar University, Bahir Dar, Ethiopia; 3 School of Public Health and Tropical Medicine, Tulane University, 1440 Canal St, New Orleans, LA 70119 USA; 4 International Potato Center, Addis Ababa, Ethiopia; 5 Agriculture for Nutrition and Health Research, International Food Policy Research Institute, Addis Ababa, Ethiopia; 6 School of Human Ecology, University of Wisconsin, Madison, USA

**Keywords:** Malnutrition, Nutrition sensitive, Agriculture, Health systems research

## Abstract

**Background:**

Nutrition-sensitive agriculture is an effective multi-sectoral approach to address the underlying causes of malnutrition. However, successful implementation requires the involvement of different sectors to jointly plan, monitor, and evaluate key activities, which is often challenged by contextual barriers. Previous studies in Ethiopia have not adequately explored these contextual barriers. Hence, the current study aimed to qualitatively explore the challenges to joint planning, monitoring, and evaluation for nutrition-sensitive agriculture among sectors in Ethiopia.

**Methods:**

A qualitative exploratory study was conducted in Tigray and Southern Nations, Nationalities, and Peoples (SNNP) of Ethiopia regional states in 2017. Ninety-four key informants were purposively selected from government agencies primarily in health and agriculture, from local (kebele) to national levels, and ranging from academic organizations, research institutions, and implementing partners. Researchers developed a semi-structured guide and conducted key informant interviews which were audiotaped, transcribed verbatim in local language, and translated to English. All transcriptions were imported into ATLAS.ti Version 7.5 software for coding and analysis. The data analysis followed an inductive approach. Transcriptions were coded line by line; then similar codes were grouped into categories. Subsequently, non-repetitive themes were identified from the categories using thematic analysis methodology.

**Results:**

The following themes were identified as challenges that hinder joint planning, monitoring, and evaluation to link nutrition to agriculture: (1) limited capacity, (2) workload in home sector (agriculture or nutrition), (3) lack of attention to nutrition interventions, (4) inadequate supportive supervision, (5) problematic reporting system, and (6) weak technical coordinating committees.

**Conclusions and recommendations:**

Gaps in human and technical resources, limited attention from different sectors, and absence of routine monitoring data hindered joint planning, monitoring, and evaluation activities for nutrition-sensitive agriculture in Ethiopia. Short-term and long-term training for experts and intensification of supportive supervision may address gaps in capacity. Future studies should address whether routine monitoring and surveillance in nutrition-sensitive multi-sectoral activities provides long-term improvement in outcomes.

## Background

Malnutrition contributes to the death of 45% of children under age five globally, while around two billion individuals worldwide are suffering from food insecurity [1, 2]. Nutrition-sensitive agriculture programming is recognized as an important means to address the underlying determinants of under-nutrition by maximizing the positive impact of the food system on nutrition outcomes for mothers and children [3]. Evidence from low-income countries points to the importance of multi-sectoral collaboration across stakeholders to address the multi-factorial determinants of under-nutrition [4–8]. Despite improved awareness of the importance of nutrition and agriculture in recent years, there is still little understanding of how to carry out effective implementation of the interventions related to policy objectives [9]. In this regard, joint planning, monitoring, and evaluation is an important step forward to link nutrition to agriculture across sectors [8–12].

Malnutrition has been identified as a target public health problem among children and women in Ethiopia [13, 14]. Improving collaboration across the nutrition and agriculture sectors in planning, monitoring, and evaluation to link nutrition and agriculture is crucial for the country to address the continued high prevalence of malnutrition. Ethiopia acknowledged the role of multi-sectoral coordination for nutritional interventions in the National Nutrition Program I of 2013, National Nutrition Program II of 2016, and Seqota declaration of 2015, which all firmly recognized addressing malnutrition as a key national priority at the policy level [15]. Recognizing the crucial role of joint action in planning, monitoring, and evaluation for nutritional interventions, the country articulated the need to strengthen collaboration of institutions and organizations from the grassroots to the national level. Accordingly, the health, agriculture, education, water, and other sectors are expected to jointly plan, monitor, and evaluate relevant activities to address severe public health problems [16]. Particularly, the National Nutrition Program II has aimed to implement nutrition-sensitive agriculture across sectors, including health, agriculture, water resources, social protection, and others as a strategic objective with specified targets and initiatives. Incorporating the lessons from the previous plan (NNPI) [16], the program calls for the need to improve community, workforce, institution, and systemic-level capacity to implement nutrition-sensitive agriculture [15]. Approaching these matters jointly helps sectors have clear mandates, high-level commitment, adequate authority, and responsibility to sufficiently respond to nutrition needs. It is considered among the most effective approaches to tackle the underlying causes of malnutrition [5, 17, 18].

However, efforts to promote this approach of joint planning, motoring, and evaluation (PME) are challenged by multiple attitudinal and organizational constraints. Previous studies identified challenges to nutrition-sensitive agriculture, such as poor awareness, poor political will and commitment, low financial allocation, and limited human resource [5–7, 17–19]. The National Nutrition Program (NNP) and the nutrition-sensitive agriculture Strategic Plan of Ministry of Agriculture and Natural Sciences (MoA&NS) to promote production and consumption of bio-fortified crops, like vitamin A-rich orange-flesh sweet potatoes (OFSP). As a part of the effort, the International Potato Center (CIP) and its partners implemented projects aimed to promote orange-flesh sweet potatoes (OFSP) in Tigray and Southern Nations, Nationalities, and Peoples’ (SNNP) regions of Ethiopia since 2011. However, lack of institutionalization, poor cross-sectors coordination, capacity-related factors, and poor attention to consumption of such products affect the effort and the lessons from the projects call for systemic diagnostic study on detailed challenges for collaboration in nutrition-sensitive agriculture in Ethiopia. Promoting production and consumption of nutrient-rich agricultural products requires multi-sectoral collaboration, with agriculture and health sectors at the core of it, to jointly plan, monitor, and evaluate nutrition-sensitive activities. To support this vision, steering committees that consist of leaders of sectors and technical coordinating committees that consists of technical experts from each sector have been established at national, regional, woreda, and kebele levels to support the activities, including joint planning, implementation, and evaluation through close supportive supervision. However, previous studies in Ethiopia do not adequately explored the array of challenges that hinder collaboration to jointly plan, implement, and evaluate nutrition-sensitive agriculture.

Understanding operationalization of the challenges across the structural levels, from frontline level to national level, would be a step toward realization of the opportunity for interventions that link nutrition to agriculture. Therefore, the current study aimed to explore the challenges that prevent sectors from undertaking joint PME linking nutrition to agriculture using the specific example of implementation of an intervention related to production and consumption of orange-flesh sweet potatoes (OFSP) in Tigray and Southern Nations, Nationalities and Peoples’ (SNNP) regions of Ethiopia.

## Methods and participants

### Study setting

This study was undertaken in Tigray and SNNP regions where International Potato Center (CIP) and partners implemented projects aimed at promoting production and consumption of nutritious OSFP in both regions of Ethiopia. The regions are among areas with a high prevalence of maternal and child chronic and acute malnutrition according to recent indicators. Despite rapid agriculture led economic progress in Ethiopia over the last two decades, malnutrition continues with high levels of stunting (37%), underweight (21%), and wasting (7%) [13]. Recently, there has been a national movement toward developing policy, strategies, and programs to address severe public health problems through involvement of multiple sectors. The current study was part of a larger systems diagnostic study on implementation challenges and opportunities for nutrition-sensitive agriculture interventions in Ethiopia. Here, the current study specifically targeted challenges that hinder joint planning, monitoring, and evaluation of activities that link nutrition to agriculture in the context of Ethiopia. This study was conducted from September to October, 2017.

An exploratory qualitative study utilizing key informant interviews (KII) was conducted to understand the challenges for joint planning, monitoring, and evaluation across sectors to link nutrition with agriculture in Ethiopia. Thematic analysis was utilized to assess findings throughout.

### Sampling and data collection

The primary method of data collection was in-person, in-depth KII undertaken with a purposively selected sample of key informants chosen by expertise, administrative position, work experience, and year of stay in the position related to the topic (6 months and above). During desk review, researchers identified and framed the existing coordinating platforms and key stakeholders in nutrition-sensitive agriculture in the regions (see Table 1). Participants were experts and administrative representatives from the Federal Ministry of Health, five from Agriculture and Natural Resources, six from Regional Health and Agriculture Bureaus, ten from Health and Agriculture zonal offices, and twenty four from Health and Agriculture woreda offices. Six health extension workers at health posts, six heads of Farmers’ Training Centers (FTCs), and ten experts working in the centers were also part of this study at kebele level. In addition, four participants were recruited from Agricultural Technical and Vocational Education and Training centers (ATVETs), while two were from Health Science Colleges. Implementing partners operating in nutrition-sensitive and -specific sectors at national and regional levels were also included in this study (see Table 1). Participants were approached before the actual interview and asked for convenient time and place to reduce possibility of interrupting the interview for office works. Each interview was conducted in place where privacy of the participants was kept and recording was possible with minimal disturbance. Each interview lasted a minimum of 45 min and the filed notes and the recorded audio were labeled, transferred into personal files that deny access to others than the investigators.

**Table 1:** Categorization and recruitment of study participants

Level	Type of institution	Number
National (*n* = 05)	Federal Ministry of Agriculture and Natural Resources	01
	Federal Ministry of Health	01
	National Nutrition Coordination Body (NNCB)	01
	Nutrition Case team Members in Ministry of Agriculture and Natural resource	01
	Other coordinating platforms as identified from desk review	01
Regional (*n* = 06)	Regional Bureau of Agriculture and Natural resource	02
	Regional Bureau of Health	02
	Regional Nutrition Coordinating Body	02
Zonal (*n* = 10)	Zonal Bureau of Agriculture and Natural resource from 2 zones	04
	Zonal Bureau of Health from 2 zones	04
	Zonal Nutrition Coordination Platform	02
Woreda (*n* = 24)	Woreda office of Agriculture and Natural resource from five target woreda each	10
	Woreda Office of health Tigray	10
	Woreda-level agri-nutrition coordination platforms	04
Kebele (*n* = 20)	Kebele Bureau of Agriculture and Natural resource offices (Development Agriculture)	10
	Kebele Health offices (Health extension workers (HEWs)	10
Training institutions (*n* = 12)	Agricultural Technical and Vocational Educational and Training centers (ATVETs)	04
	Health Colleges	02
	Farmer Training centers (FTCs)	06
Implementing institutions (*n* = 17)	Partners and research organizations	17*
Total		**94**

^*^ = Save the children, REST, World vision, FAO, MUMs for MUMs, SURE, EPHI, and others

A pretested semi-structured guide was used for the key informant interviews. The tool was developed by researchers following review of literature, government policy documents, existing platforms, possible stakeholders, and existing strategies in agriculture and health sectors to link nutrition with agriculture. The review provided a broader study on systems diagnostic of implementation challenges and opportunities for nutrition-sensitive agriculture interventions in Ethiopia which informed the interview guide. The broader study consisted of basic diagnostic principles including joint planning, monitoring, and evaluation. The current manuscript specifically focused on challenges to PME jointly done across sectors. The questions mainly asked participants awareness and perception on the necessity of integrating agriculture to nutrition and health, specific activities that their institution is doing to link agriculture and nutrition with specific examples (if any), efforts the institution is doing to jointly plan, implement, and evaluate activities that link agriculture to nutrition, and the challenges their institutions faced. Probes were employed for each questions and emerging concepts were incorporated in to the tool for the successive interviews.

#### Trustworthiness

The investigators considered their prior conceptions, expectations, and experiences to counteract potential bias during data collection, transcription, and analysis. Data from key informants at different levels were triangulated for congruence and variation. During the in-depth interview, participants were probed using follow-up questions for points that needed clarification, completion, and depth. Data collection and analysis were undertaken concurrently. Preliminary analysis of collected data was done to incorporate emerging insights and views into the interview guide. Investigators also conducted debriefing sessions on daily basis to enhance trustworthiness of the data.

### Data analysis

Thematic analysis as outlined by Braun and Clarke provided the analytic strategy [19]. Each audiotaped interview was listened to repeatedly, transcribed verbatim, and imported into ATLAS.ti qualitative data analysis software version 7.5 (ATLAS.ti Scientific Software Development GmbH, Berlin, 2015) for coding and analysis. Field notes and investigator memos were also linked to files in the software to assist analysis. Text was coded based on its relevance to the central topic of inquiry. Two investigators openly coded the generated transcripts using an inductive approach. The two investigators checked consistency in coding on a daily based during peer debriefing. In the case of inconsistency, a third investigator was called for achieving shared understanding of the coding. Then, the two investigators assessed the revised set of codes for text congruency and linkage using axial coding. Consequently, similar codes were systematically categorized and categories were labeled. Finally, the labeled categories were transformed into candidate themes and final, non-repetitive themes. The investigators also engaged in debriefing and discussions during analysis to assure emerging themes and results were grounded in the data and the dimensions of the challenges were well captured. Description of the themes, the sub-categories under each theme, and illustrative quotes supporting the descriptions were used in writing the result.

### Ethical considerations

The institutional review board (IRB) of the College of Health Sciences at Mekelle University approved the protocol of the study. Written consent was sought from each participant after the objectives and the purposes of this study were explained and confidentiality was maintained.

## Results

### Current practice

Participants at kebele (smallest administrative unit) level (heads of farmer's training centers, development agents for agriculture, and health extension workers) reported that they plan activities pertaining specifically to their domain separately, and then compile and review these together. Consequently, these sectors try to implement their own planned activities at their domain level and review their performance together twice per month. The kebele leader, kebele stream committee, and kebele command post were frequently reported agents that organize joint PME. Based on the performance, the organizing agent ranks the sectors, acknowledging successes, and initiating corrective measures for failure. The agent also considers re-planning of activities that were not successful. Participants also mentioned that there is direction from the woreda level (administrative level above kebele, which is equivalent to district) to jointly plan, monitor, and evaluate activities that link nutrition to agriculture. A pattern analysis of the activities that the institutions were doing to link agriculture to nutrition, institutional, and systematic activities to jointly plan, implement, and evaluation was relatively stronger at kebele level than its higher level. One expert stated:

*“The activities related to nutrition would be planned together with the HEWs and the education sector. The planning would be debriefed and reviewed by the sectors. Then after, there would be joint review meeting and discussion to monitor the performance. The reason behind the lower performance will then be identified and the re-planning of the activities that need compensation of implementation would be undertaken”* [DA-FTC-Tigray Region]

Among the nutrition-related activities included in the kebele command post plan were the following: community based nutrition, safety net programs, provision of high nutrient cereals and crops to farmers, provision of safe drinking water, and awareness creation around programs. Frontline workers cascaded the planned activities to local development armies (women development armies, farmer development armies, and youth development armies) for implementation.

At woreda level, steering and technical committees have been established to coordinate nutrition-related activities, though weakness in these has been identified. Some woredas reported yearly interface of different sectors for performance review, joint supervision to kebeles, reporting on exchanges among sectors, and provision of joint training to frontline professionals. Most of these activities were organized by non-governmental organizations or partners. Participants also mentioned efforts to strengthen joint PME at regional and national levels, including events, like workshops and performance review meetings (though these were reportedly irregular and with low accountability). A key informant from Sidama zone reflected,

*“There is no coordinated work with the implementing partners. We don’t have joint planning, implementation and monitoring and evaluations. The partners are doing each activity alone, which could be cosnidered as a bottleneck that I noted regarding OFSP*” [MCH expert, SNNP].

### Challenges to joint planning, monitoring, and evaluation

The following themes emerged as themes for challenges to jointly plan, monitor, and evaluate activities that link nutrition to agriculture: (1) limited capacity, (2) workload of individual sectors, (3) lack of attention to nutrition interventions, (4) poor supportive supervision, (5) poor reporting system, and (6) weak technical coordinating committees.

Participants reported limited capacity among personnel working at kebele, woreda, and regional levels, which hinders joint PME to link nutrition and agriculture. Limited training, absence of manuals, and guidelines on specific activities all reportedly lead to the low capacity. In addition, participants reflected that there is absence of post-training follow-up. Once capacity building interventions, such as training, were provided to strengthen the linkage, there was no follow-up on effectiveness. Particularly, one agricultural development agent described it this way:

*“For some activities to be implemented in linkage, all experts in the sectors need to be trained on the issue to have a common level of understanding regarding the issue. The DA should be trained together with the health extension worker or the teacher” ****[****DA-Tigray region****].***

Participants at woreda, zonal, and regional levels also reported that there were no nutrition professionals in the sectors, such as health and agriculture, to coordinate the activities. They assumed that the professionals would help steering committees give focus to the activities.

#### Workload

Frontline workers frequently reported that workload prevents them from engaging in joint PME activities to link nutrition to agriculture. Engagement in competing tasks that belong to each sector was also reported and deemed more time sensitive at the kebele level, causing nutrition-related activities to be sidelined. This was reported by both kebele and woreda-level key informants in particular.

“There was a closer monitoring by the woreda coordinators from both agriculture and health sectors. But, recently, it becomes weaker. You know there are also other competitive, mandatory and urgent tasks to be performed first. Hence, the nutrition related activities would remain suppressed. Then, the review meeting, evaluation and monitoring gets weak” [*Woreda Agriculture office-Tigray Region*].

A key informant at kebele level also reflected his view as

*“Plan versus implementation always varies at kebele level and task overload is among the main frequently cited. There is load of tasks among the experts and most of the activities in agriculture are seasonal and must do with the time set” [DA-FTC-Tigray region****].***

Partners also complained that sector heads and representatives are mostly busy in other competitive activities and meetings, which makes it difficult for them to get involved.

#### Lack of attention to nutrition interventions

Participants also described that individuals from different sectors and committees did not provide sufficient time or attention to review the performance of nutrition-related activities. One example of this was not including nutrition topics in their supervision checklist. An expert stated it this way:

“*I do not think that the nutrition related activities are too difficult to undertake but there is no attention given to it. We only seek collaboration for activities like sanitation. Similarly, no focus is given to nutrition in the kebele performance review meeting*” [HEW-Health Post, Tigray Region].

Participants described lack of collaborative work style, punctuality, and adherence to agreed working principles as some of the constraints that prevented to joint PME activities. Regional-level participants reported barriers, such as accountability to the activities, irregularity in the meetings, non-specific scope of the collaboration, and passive involvement of members. A nutrition coordination body member stated it this way: “There may be monitoring and evaluation activities at each sector separately. However, there are no joint activities aimed to monitor the agriculture-nutrition interventions” [Nutrition expert, Tigray regional health bureau].

A regional expert also reflected this:

*“The committee is too weak from the higher to the lower level. Works that need to be done by coordination and individually are clearly laid out and I think what is remaining is the coordinated work at the ground level”* [Agriculture higher expert, Tigray region].

#### Inadequate supportive supervision

Although the sectoral collaboration calls for close supportive supervision by technical coordinating committees and the steering committees from higher structural levels, participants from Kebele frontline reflected that immediate supervisors do not supervise and support the activities undertaken to link nutrition with agriculture. The activities are not included in the supervisory checklist and little or no attention is given to them. Participants reported that there is a lack of follow-up, no request for report, and no feedback for the activities. Similarly, woreda-level key informants also underlined that there is no clear direction and supportive supervision from regional-level sectors on the issue. The view is captured in the following quote:

*“Our problem is, agriculture workers [supervisors] come from higher level and ask us only the activities that they assume belong to agriculture, ignoring nutrition related activities. At this moment, we feel sad and focus on our task*s only. This declines our intent to be involved in the linkage”[DA-Kebele-Tigray region].

A zonal coordinator also explained it this way:

“At lower levels, awareness creation regarding nutrition sensitive agriculture has been started. For example, it has been started in Ofla, and Agaw Woredas, though not strong. We haven’t supported our Woredas and I haven’t heard such support from the region on this regard” [Zonal Agriculture extension Coordinator, Tigray region].

Lack of post-training follow-up by supervisors was also captured in the following quote:

*“[…] we are providing training on how to do things [related to nutrition] better but while they go back to community, the supervision we made is very weak. We do not monitor the changes and no supportive supervision as well”*(Zonal expert, SNNP).

#### Problematic reporting system

Participants, mainly key informants from partner organizations, also reported gaps related to the reporting system hindering joint PME. Lack of monitoring and evaluation tools, no joint data management system, lack of continuous, and clear common reporting system were identified. In succession to uncovering the lack of jointly developed tools, a key informant remarked on the absence of a joint data management system:

*“One of the challenges [for joint PME] is that there is no joint data management system for the thirteen signatory sectors about nutrition issues for their joint evaluation and accountability”* [SURE project staff, Tigray region].

Partners working in the regions also underlined the inconsistency of the reporting system by stakeholders. A key informant stated this:

*“One of the challenges is lack of continuous, clear and common reporting [regarding activities that link nutrition to agriculture], among stakeholders. Each stakeholder has its own reporting system with low data consistency and completeness”*[World Vision project staff, Tigray region].

Similarly, the absence of tools appropriate to nutrition-sensitive agriculture work emerged as a challenge for reporting. An expert from SSNP stated it this way, “The second challenge is that lack of jointly developed M&E activitiy tools from the 13 signatory sectors from their ministries' level to the kebele level” [Nutrition expert, SNNP].

#### Weak technical coordinating committees

Participants reflected that the steering and technical committees at kebele, woreda, and regional levels are weak in organization. The committees often have no plan, and performance review meetings are not regular and consistent. Participants reported an absence of commitment to implement activities, conduct supervision, and review meetings, and to adhere to planned schedules. An expert stated this:

*“Of course there are directions that we need to link the agriculture with health and water hygiene. However, there is no specific plan pertaining to the issues”* [DA-Kebele, Tigray region].

Key informants from woreda, zonal, and regional levels reported that the concept of joint PME to link nutrition to agriculture was still new and underdeveloped.

*“We haven’t yet started to evaluate in this way [jointly] but we have oriented all sectors and prepared to do so. I know we are late to organize it”* [Woreda nutrition coordinator,Tigray region].

*“It is too early to evaluate the impact of the intervention [OFSP] on nutrition. We have started to implement interventions but we haven’t evaluated their impact on nutrition yet”* [Woreda health office-Tigray region].

The current study also revealed that joint planning and reporting among sectors seems to be limited to emergency cases. A zonal nutrition expert stated,

*“We have been working together [health, nutrition, and agriculture] during an emergency case only. As a zone we do not have such integration plan or system with agricultural sector to link the health sectors. We only meet during an emergency and during report preparation”[Zonal Nutrition focal person, SNNP].*

A regional expert also reflected,

*“I do not remember an activity related to nutrition that my institution has involved in joint monitoring and evaluation. The activities related to nutrition are recently included to the institution following the Seqota declaration in the September 2017. Our institution has not engaged in such monitoring and evaluation related to nutrition”* [Regional Women's Affair Bureau staff, Tigray].

## Discussion

Overall, our study identified that systematic activities that facilitate joint PME occurred more at lower administrative level (kebeles) rather than at higher levels. Sector staff at kebele level develop their own plans and bring their plans for joint review, implementation, and performance review. The sectors are currently working in collaboration on nutrition-sensitive activities but not at a consistent level. Facilitated joint activities on nutrition-sensitive agriculture were reported between partners at woreda level. The main challenges for joint planning, monitoring, and evaluation of nutrition-sensitive agriculture clustered around personnel capacity, workload of sector personnel, weak technical committees/task forces, lack of attention given to nutrition activities, inadequate supportive supervision, and problematic reporting system.

In line with our findings, a review of evidence identified lack of expertise in nutrition and poor cross-sectoral knowledge as major obstacles to jointly implement nutrition-sensitive interventions in Malawi, Nepal, and Sierra Leone [9]. Other studies also indicate limited strategic, technical, and operational capacities as a result of an insufficient number of nutrition officers, staff turnover, lack of knowledge of policy documents, inadequate training for frontline workers [17, 20–22], and other capacity-related constraints [23–26].

Moreover, the current study illustrates that the need to link agriculture to nutrition is an overlooked concept by experts working across different levels and sectors. This insight is aligned with a study on mid-level actors for nutrition-sensitive agriculture in Ethiopia, which shows that the long-term emphasis on production neglected concerns around nutrition and dietary needs [27]. This implies the need to proactively involve local stakeholders to improve cross-sectoral knowledge on nutrition. To achieve this end, some countries have involved nutrition champions [28, 29].

Workload also emerged as an often reported challenge to joint PME in linking nutrition to agriculture. However, it may also be a reflection of the lower priority assigned to the activities that link nutrition to agriculture compared with other day to day activities. This issue is likely also a reflection of the lack of human resources responsible for coordination of these activities [20]. A previous study from South Asia and East Africa indicates that copious meetings within individual sectors restricted the potential synergies in creating joint platforms in nutrition-sensitive agriculture [17]. In Ethiopia the heavy workload among health extension workers may also prevent the implementation of nutrition-related activities [26]. This may imply that the National Nutrition Plan might need to come up with an innovative approach to strengthen multi-sectoral collaboration that accounts for the heavy workload among each sector’s front line workers.

Insufficient attention and priority given to nutrition were also recognized as a challenge for joint PME in linking nutrition to agriculture. For other sectors, the issue of nutrition is considered secondary to their “main activities.” Similarly, previous studies from Nepal and Pakistan show that many stakeholders are unaware of their role to address nutrition-related problems and thus exhibit poor commitment to joint PME [17]. In many areas, the issue of joint PME in linking nutrition to agriculture is considered to be the responsibility of the health or agriculture sectors only, whereas most of the activities are limited to emergency issues related to food security [20]. Despite political will and government priority assigned to nutrition-sensitive agriculture [27], the day to day responsibility for realizing this goal does not benefit from support of local authority, power, and resources, including budget [5, 17, 20, 23, 26]. This might be backed by the skewed attention of agricultural sector toward productivity and the health sector toward other immediate risk factors for diseases, rather than nutrition. Participants also reported that activities related to nutrition-sensitive agriculture programs, workshops, and trainings are primarily funded by donors, which is congruent with a previous study from East African countries[18]. This could be an indication of the low priority and attention given by the local governments. Lack of awareness regarding nutrition and viewing nutritional activities as only the responsibility of the health sector was also previously reported in Ethiopia [25, 26]. A previous study also found that government officials have limited interest in nutrition and that agricultural officers have less understanding of how to operationalize it [22]. Agricultural officers in the country may restrict their primary goal to increasing cereal production, while women’s affairs officers assume a lesser role than expected [26]. In addition, the current study identified poor support from immediate supervisors and across the hierarchy. In particular, frontline workers highlighted the limited attention and support provided by immediate supervisors. A study from Mozambique identified lack of coordination in planning and implementing activities, as well as poor accountability and irregular monitoring/evaluation of the activities, that hampered multi-sectorial collaboration for nutrition, in line with the current findings [9]**.**

The current study also highlighted poor reporting systems as a challenge to jointly plan, monitor, and evaluate efforts to link nutrition with agriculture. The 2008 Ethiopia national nutrition strategy also articulated the need to strengthen continuous and regular monitoring and assessment for nutritional interventions and their outcomes [16]. The current study found poor reporting systems continued to be a challenge for joint PME. Similarly, an earlier study in Malawi, Mozambique, Senegal, and Sierra Leone revealed poor reporting systems as a challenge for multi-sectoral collaboration in nutrition which was evident in the lack of data, no regular collecting of data, absence of regular monitoring and evaluation, and absence of nutrition indicators in checklists [9]. Even projects that include explicit nutrition components lack nutrition-focused indicators in monitoring and evaluation while inappropriate or inadequate use of existing data was also a concern noted in Eastern African countries through studies there [17, 20]. This may imply the need to either introduce or integrate data management systems sensitive to multi-sectoral collaboration at all structural levels. Weakness of the committees was also noted by informants even though Ethiopia’s national nutrition strategy in 2008 sought to establish and strengthen nutrition coordinating bodies at all levels [16]. This low commitment may be a function of a low level of priority assigned to the activities.

## Strengths and limitations

The extensive review undertaken to develop the tool and the qualitative exploration approach enabled investigators to understand the challenges in depth and how they function. Inclusion of participants from a variety of positions and levels also helped uncover the challenges for collaboration. However, the study was not without limitations. One limitation is that this study employed only one qualitative method, key informant interviews, which may restrict diverse opinions as it is limited by the purposive and non-probabilistic sampling. Additionally, the study is cross-sectional in nature, and conducted at one point in time, which does not allow for longitudinal understanding of the joint planning, monitoring, and evaluation process for key activities relevant to link agriculture and nutrition.

## Conclusions and recommendations

Although there is much energy and support for the formulation of policy and strategy for joint planning, monitoring, and evaluation to link nutrition with agriculture, it is operationally challenged by limited human and technical resources, limited attention given to nutrition, and absence of regularly collected data on nutrition-sensitive activities. Coordinating bodies were found to be weak in terms of capacity and attention, with no clear means of ensuring accountability. Training institutions should consider training programs to close the gap in technically capacity for nutrition. Additional capacity building activities could include short-term training and supportive supervision to improve cross-sectoral knowledge and skills to coordinate and implement nutrition-sensitive agriculture programming. More importantly, joint activities across sectors at different levels of benefit from regular collection and analysis of data on key indicators of nutrition-sensitive agriculture.

## Data Availability

The tools used for this study and the data that support the findings in this study are fully available on request from the authors.
